# A meta-review of psychological resilience during COVID-19

**DOI:** 10.1038/s44184-022-00005-8

**Published:** 2022-07-05

**Authors:** Katie Seaborn, Kailyn Henderson, Jacek Gwizdka, Mark Chignell

**Affiliations:** 1 Department of Industrial Engineering and Economics, Tokyo Institute of Technology, Tokyo, Japan; 2 Department of Mechanical and Industrial Engineering, The University of Toronto, Toronto, ON Canada; 3 School of Information, The University of Texas at Austin, Austin, TX USA

**Keywords:** Psychology, Health care, Public health, Quality of life, Scientific community, Research data

## Abstract

Psychological resilience has emerged as a key factor in mental health during the global COVID-19 pandemic. However, no work to date has synthesised findings across review work or assessed the reliability of findings based on review work quality, so as to inform public health policy. We thus conducted a meta-review on all types of review work from the start of the pandemic (January 2020) until the last search date (June 2021). Of an initial 281 papers, 30 were included for review characteristic reporting and 15 were of sufficient review quality for further inclusion in strategy analyses. High-level strategies were identified at the individual, community, organisational, and governmental levels. Several specific training and/or intervention programmes were also identified. However, the quality of findings was insufficient for drawing conclusions. A major gap between measuring the psychological resilience of populations and evaluating the effectiveness of strategies for those populations was revealed. More empirical work, especially randomised controlled trials with diverse populations and rigorous analyses, is strongly recommended for future research.

## Introduction

The COVID-19 pandemic has disrupted many aspects of life at a global scale. Mental health and psychological well-being have subsequently emerged as key research foci in healthcare and public health during the pandemic^1–4^. Most countries have endorsed interventions with known or foreseen effects on psychological well-being, such as social distancing, physical isolation, and self-quarantine. Given what is already known about the relationship between mental health and psychological interventions^5^, this has further motivated questions on the assessment, management, and prevention of negative psychological outcomes^1–3,6–8^. *Psychological resilience* plays an essential role in times of crisis. As a behavioural characteristic, it can be framed as positive adaptability: the ability to “bounce back” when confronted with unusual and negative circumstances involving adversity, stress, and trauma^9,10^. Psychological resilience may be affected by socio-economic status^11^, cultural factors^12^, and other sources of influence. In pandemics, psychological resilience may dramatically affect outcomes. External offerings, such as social support systems, may reduce levels of depression^1^, while internal orientations related to psychological stress^2^, coping skills^2^, positive mood^7^, and positivity, especially “finding the good in the bad,”^9^ are all facets of psychological resilience that subdue or prevent negative outcomes. The extent to which it is achieved, and how, may be a fundamental determinant of a population’s ability to combat mental health difficulties resulting from stressors related to the COVID-19 global pandemic.

A meta-review is a standard way of assessing the state of affairs. Meta-reviews, also termed umbrella reviews or overviews of reviews, are systematic reviews of extant review work that aim to achieve clarity and consensus on a specific research question or topic while considering factors such as review quality and bias^13,14^. Intended beneficiaries are decision-makers, the academic community, and the public. Meta-reviews synthesise systematic reviews and meta-analyses of primary studies, which typically represent the highest achievable level of evidence^13^. As such, assessing the quality of the body of review work is a key component of meta-reviews^13^. However, the COVID-19 pandemic has resulted in a unique set of circumstances. Indeed, the ongoing, pressing need for answers has led to a large number of submitted manuscripts, as well as greater leniency in publishing criteria^15^. Emerging from this “paperdemic” are crucial questions regarding scientific integrity during COVID-19^15^. The collection of review work on psychological resilience may be subject to the same pressures of time and demand. Yet, as indicated by citation counts and media coverage, this work is being relied upon to inform our understanding of the situation and make public health decisions. A rigorous evaluation is necessary to reach consensus for healthcare governance and identify current inadequacies that must be accounted for in future editorial policies and publishing requirements.

This meta-review addresses an urgent need to both assess what is known about psychological resilience during COVID-19 and appraise the quality of research and review work being conducted on this topic. Our research objectives were: (RQ1) to summarise the nature and quality of this body of work and (RQ2) to derive a consensus on strategies implemented to evaluate, maintain, and cultivate psychological resilience throughout the COVID-19 pandemic. While our objective was to provide a reliable overview of the review work along with the means for building knowledge and taking action, we were largely limited by the state of the literature. In short, we cannot offer strong evidence for or against the strategies gathered across the corpus of survey work. Indeed, the severe limitations in this body of work are alarming and undermine the recommendations offered by specific reviews, however highly cited. We map out a series of psychological resilience factors, measures, and strategies gathered from these reviews that, while having potential validity, urgently need high quality empirical work on their efficacy within the context of the COVID-19 pandemic.

## Results

### Review sample and characteristics

**Fig. 1:**
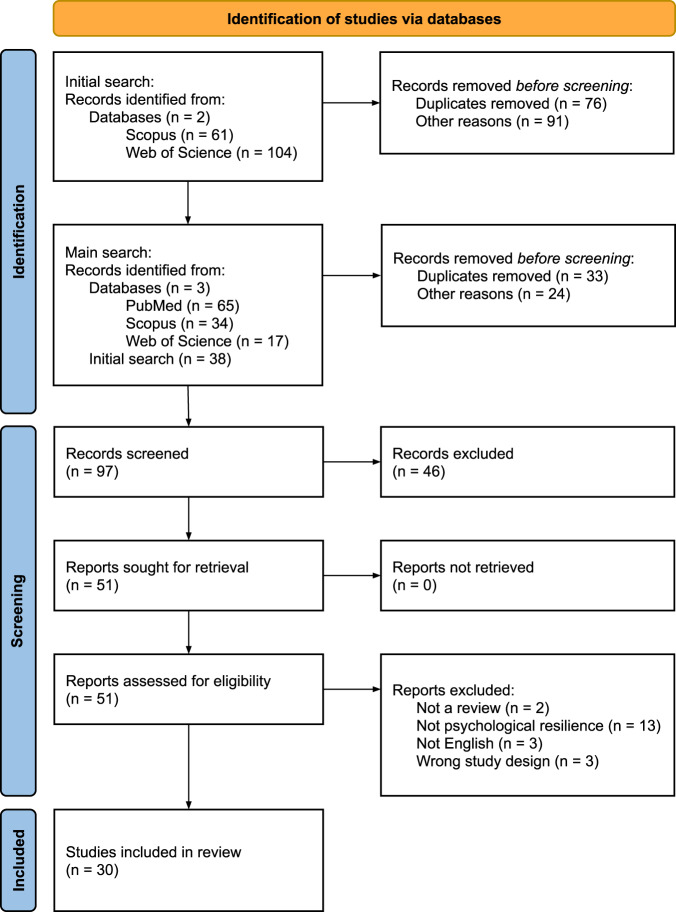
PRISMA flow diagram of study inclusion. PRISMA flow chart showing study inclusion and exclusion at the identification, screening, and included stages. The identification stage featured an initial search of two databases and a main search of three databases. The screening stage involved screening records, retrieving reports, and assessing their eligibility. Of these, 30 were included in the final stage.

From an initial total of 281 reviews retrieved across three databases in two phases, 97 were screened and 30 were selected for analysis (Fig. 1). Excluded reviews and reasons for their exclusion are presented in Supplementary Table 4.

General characteristics of the included reviews are presented in Supplementary Table 5. Study characteristics were extracted from the 30 included reviews, all of which were published in 2020 or 2021. The review types included narrative (7), rapid (8), systematic (2), scoping (4), mini (1), and mixed methods (1). Only ten (33%) reviews used protocols: four pre-registered with PROSPERO, two with OSF (one in parallel), one with Cochrane Reviews, and three available but unregistered. Of these, one was available from the authors upon request, one was uploaded to an institutional website, and one explained “any discrepancies from the study as planned (and, if relevant, registered)”^11,16^. Three reviews were highly cited (50 or more citations) but had no registered protocol and were deemed of low quality; three had registered protocols and were highly cited but of low quality; and five highly cited reviews, deemed of sufficient quality, had no registered protocol.

Participants were from the general population (all ages), as well as subset populations such as individuals working in healthcare (e.g., nurses, doctors, medical staff, social workers, etc.). Specific settings included hospitals, clinics, medical centres, and workplaces. Specific contexts mostly pertained to specific outbreak and pandemic situations, such as SARS, COVID-19, Ebola, H1N1, and MERS (Table 1). Twenty-five (83%) studies reported the number of databases searched, which ranged between 1 and 14 (M = 4.84, SD = 2.56, MD = 4, IQR = 3), with the earliest search being carried out on November 17, 2019, and the latest search on March 15, 2021. Twenty-three reviews (77%) reported the number of studies included, which ranged from 2 to 139 (M = 36.65, SD = 32.09, MD = 25, IQR = 31). These included qualitative and quantitative study designs: cross-sectional (surveys, observational), longitudinal, randomised controlled trials (RCTs), descriptive, cohort (prospective, retrospective), interviews, reviews, case-control, and mixed-methods. These studies were conducted in and across six continents: Asia, Africa, North America, South America, Europe, and Australia. Frequent countries of origin of the studies included China, UK, USA, Canada, India, Hong Kong, Italy, and Taiwan. Many of the outcomes reported pertained to the psychological and mental health impacts, e.g., anxiety, stress, depression, posttraumatic stress disorder (PTSD), insomnia, of COVID-19, and risk factors for these impacts.

**Table 1:** Contexts in which measures were tested.

Context	Total reviews and citations
COVID-19	9^20–24,46–49^
COVID-19 and other pandemics	15^16,18,19,27,28,50–59^
Not COVID-19	1^17^
Not reported	5^29,60–63^

### Risk of bias across reviews

Full details of the risk of bias assessments are presented in Table 2. The mean SANRA scores for qualitative reviews was 0.74 (SD = 0.12) and the mean JBI score for all other reviews was 0.78 (SD = 0.21). Based on the cut-off of 0.8, 15 reviews were determined to be of sufficient quality to answer RQ2: psychological resilience strategies.

**Table 2:** Risk of bias assessments.

Author/s (year)	Average SANRA score (%)	Average JBI score (%)
Jans-Beken et al.^50^	0.71	
De Kock et al.^46^		0.89
Schwartz et al.^60^	0.79	
Blanc et al.^51^		0.09
Hooper et al.^17^		0.81
Giorgi et al.^20^	0.92	
Wright et al.^61^		0.4
Varghese et al.^47^		0.95
Sterina et al.^52^		0.79
De Brier et al.^53^		0.82
Sirois and Owens^54^		0.84
Rieckert et al.^16^		0.76
Hughes et al.^48^		0.95
Pollock et al.^18^		0.95
Batra et al.^22^		0.95
Kunzler et al.^19^		0.9
Davis et al.^62^	0.67	
Chew et al.^55^		0.76
Labrague^21^		0.89
Balcombe and de Leo^56^		0.38
Gilan et al.^49^		0.9
Heath et al.^29^	0.75	
Berger et al.^57^		0.71
Ho et al.^58^		1
Muller et al.^23^		0.83
Kaur and Som^63^	0.5	
Prati and Mancini^24^		0.8
Preti et al.^27^		0.63
Seifert et al.^59^	0.79	
Etkind et al.^28^		0.74

### Measures of psychological resilience

Reviews provided 31 unique positive measures (Table 3) and 55 unique negative measures (Table 4) to assess individuals’ psychological resilience status. Most also covered risk (with respect to negative measures) and protective (with respect to positive measures) factors and status results. A total of 14 risk factors (Table 5) and 7 protective factors (Table 6) were identified. Half of the factors received a GRADE score of moderate (7/14 for risk factors and 3/7 for protective factors). Counterpoints were included where possible to highlight patterns in how factors were framed and indicate where gaps and possibilities exist.

**Table 3:** Positive measures.

Measure	Instrument
Psychological resilience	CD-RISC: Connor-Davidson Resilience Scale; Wagnild and Young Resilience Scale; Brief Resilience Scale; Baruth Protective Factors Inventory (BPFI); Resilience Scale for Adults (RSA); Brief Resilience; Coping Scale (BRCS); Dispositional Resilience Scale (DRS); Resilience Scale (RS)
Coping	Brief COPE, Ways of Coping; Coping Self-Efficacy Scale for coping; Life Orientation Test-Revised for optimism; Ways of Coping Inventory
Emotional resilience	Adolescent Emotional Resilience Scale (AERS)
Personality	Temperament and character inventory-revised (TCI-R)
Mindfulness	Freiburg Mindfulness Inventory
Optimism	Life Orientation Test-Revised for optimism
Self-efficacy	General Self-Efficacy Scale; Pandemic Self-Efficacy Scale
Social support	Social Support Rating Scale (SSRS); Multidimensional Scale of Perceived Social Support (MSPSS); Perceived Social Support Questionnaire (PSSQ); Social Support Rate Scale (SSRS); Social Support at Work (SSW)
Well-being	6-item questionnaire for life satisfaction; Professional Quality of Life for professional quality of life; Satisfaction with life scale (SWLS); Short Form 12 (SF-12); Symptom Checklist 90 (SCL-90); Symptom Checklist 90 Revised (SCL-90-R); General Health Questionnaire (GHQ-12 or GHQ-28); Short Form-36 questionnaire (SF-36)

**Table 4:** Negative measures.

Measure	Instrument
Disrupted relationships	Inventory of Interpersonal Problems (IIP-32)
Adjustment disorder	International Adjustment Disorder Questionnaire (IADQ)
Perceived stigma and barriers	Military Stigma Scale; External Stigma Questionnaire for stigma; Stigma and Barriers to Care Questionnaire
Anxiety	Generalised Anxiety Disorder Questionnaire (GAD-7); Hamilton Anxiety Rating Scale (HAM-A); Zung Self-Rating Anxiety Scale (SAS); The Van Dream Anxiety Scale; Patient Health Questionnaire-4 (PHQ-4)
Depression and suicidal ideation	Depression, Anxiety and Stress Scale (DASS-2); Centre for Epidemiologic Studies Depression Scale (CES-D); Hamilton Depression Rating Scale (HAM-D); Patient Health Questionnaire (PHQ-9); Symptom Checklist Depression Scale (SCL-20); Zung Self-Rating Depression Scale (SDS); Hospital Anxiety Depression Scale (HAD); Beck Depression Inventory (BDI); Centre for Epidemiological Studies (CES-D); Montgomer-Asberg Depression Rating Scale (MADRS)
Insomnia/sleep	Insomnia Severity Index (ISI); Athens Insomnia Scale (AIS); Fatigue Scale (FS); Insomnia Severity Index (ISI) to measure sleep disorders; Numeric Rating Scale (NRS); Pittsburgh Sleep Quality Index (PSQL) to measure sleep quality
Stress	Stress Overload Scale (SOS); Perceived Stress Scale (PSS-10); Stanford Acute Stress Reaction Questionnaire (SASR); Depression, Anxiety and Stress Scale (DASS-21); Stress Response Questionnaire (SRQ); Impact of Event Scale (IES); Acute Stress Disorder (ASD); Global Stressor Index (GSI); Self Reported Stressor and Incidence Questionnaire (SRISQ); Stressor and Incidence Questionnaire (SIQ)
Burnout	Maslach Burnout Inventory (MBI); Professional Fulfilment Index (PFI); Mini-Z Burnout Survey; Oldenburg Burnout Inventory (OBI)
Distress	Kessler K6 Distress Scale (GSES); COVID Peritraumatic Distress Index (CPDI); Huaxi Emotional Distress Index (HEI); Structured Clinical Interview for DSM-IV Axis I Diagnosis (SCID-I); Brief Symptom Inventory (BSI)
Trauma	Vicarious Trauma Questionnaire
PTSD	The Child PTSD Symptom Scale (CPSS); PTSD CheckList for Civilians (PCL-C); PTSD Self-rating Scale to Measure Posttraumatic Stress Symptoms (PTSD-SS); Primary Care PTSD Screen (PC-PTSD); Clinician-Administered PTSD Scale for DSM-5 (CAPS-5)
Loneliness	UCLA Loneliness Scale
State anger	State-Trait Anger Expression Inventory; Dimensions of Anger Reaction (DAR-5)

**Table 5:** Risk factors.

Factor type	Factor	Number of reviews (citations)	Certainty of the evidence (GRADE)	Counterpoint
Demographic	Being a woman	5^22,47–49,54^	⊕⊕⊕⊝ Moderate	
	Being younger	4^20,47,49,54^	⊕⊕⊕⊝ Moderate	
	Being a migrant worker	2^20,49^	⊕⊕⊝⊝ Low	
	Having maladaptive personality traits	2^47,54^	⊕⊕⊕⊝ Moderate	Having adaptive and positive personality traits
Proximal	Proximity to COVID-19	7^18,22,46,47,49,53,54^	⊕⊕⊕⊕ High	
Occupational	Heavy workload	4^20,23,46,47^	⊕⊕⊕⊝ Moderate	
	Working in healthcare or a caregiving role	7^22,47–49,53,54^	⊕⊕⊕⊕ High	
	Being a part-timer or untenured	1^54^	⊕⊝⊝⊝ Very low	
Social	Social isolation	6^20,47–49,53,54^	⊕⊕⊕⊕ High	Social support
Material	Inadequate PPE	4^18,23,46,47^	⊕⊕⊕⊝ Moderate	Access to PPE
Physical and Psychological Health	Pre-existing health condition	2^47,48^	⊕⊕⊝⊝ Low	
	Lack of self-efficacy	2^47,54^	⊕⊕⊕⊝ Moderate	Having self-efficacy
	Lack of coping skills	2^18,54^	⊕⊕⊕⊝ Moderate	Pre-existing coping skills
Educational	No access to training and/or intervention programmes	3^18,23,53^	⊕⊕⊝⊝ Low	Provision of training and/or intervention programmes

**Table 6:** Protective factors.

Factor type	Factor	Number of reviews (citations)	Certainty of the evidence (GRADE)	Counterpoint
Demographic	Having adaptive and positive personality traits	4^21,46,48,54^	⊕⊕⊕⊝ Moderate	
Social	Social support	7^18,21,46,49,53,54,58^	⊕⊕⊕⊝ Moderate	Social isolation
Material	Access to PPE	3^20,46,54^	⊕⊕⊝⊝ Low	Inadequate PPE
Physical and Psychological Health	Having self-efficacy	3^53,54,56^	⊕⊕⊝⊝ Low	Lack of self-efficacy
	Pre-existing coping skills	4^21,49,53,54^	⊕⊕⊝⊝ Low	Lack of coping skills
Educational	Education about COVID-19	5^18,46,48,49,54^	⊕⊕⊕⊝ Moderate	
	Provision of training and/or intervention programmes	2^53,54^	⊕⊝⊝⊝ Very low	No access to training and/or intervention programmes

### Strategies for psychological resilience

A corpus of 19 high-level strategies were gathered (Table 7). Most (17/19 or 89%) could not be given a GRADE score due to insufficient evidence, and the two remaining received very low GRADE scores. A further 15 specific training and/or intervention programmes were identified. Most were only identified by one review. The programmes were: Psychological first aid (PFA)^17–19^, trauma risk management (TRiM)^17,18^, eye movement desensitisation and reprocessing (EMDR)^17,18^, cognitive behavioural therapy (CBT)^18,20,21^, cognitive behavioural therapy for insomnia (CBTi)^18^ mindfulness-based cognitive therapy (MBCT)^21^, mindfulness-based stress reduction (MBSR)^18^, occupation therapy (OT)^18^, Motivational Interviewing (MI)^20^, resilience and coping for the healthcare community (RCHC)^17^, anticipate, plan, and deter (APD)^17^, resilience at work (RAW)^17^, mindfulness training^19,21–23^, hardiness training^21,22^, and crisis intervention^20^.

**Table 7:** High-level strategies for psychological resilience during COVID-19.

Scope	Strategy	Total reviews and citations	Certainty of the evidence (GRADE)
Individual	Control media exposure	1^20^	Insufficient evidence
	Establish work-life balance	2^20,22^	Insufficient evidence
	Maximise self-efficacy	1^19^	Insufficient evidence
	Practice self-care	3^19,22,23^	Insufficient evidence
	Practice compassion	1^20^	Insufficient evidence
	Keep up with the daily: sleep well, eat well, and exercise regularly	1^27^	Insufficient evidence
Communal	Provide social support	6^20–23,54,58^	Insufficient evidence
	Provide distractions and entertainment	2^21,23^	Insufficient evidence
	Provide spiritual or religious support	2^21,58^	Insufficient evidence
	Provide mental health support	3^19,20,23^	Insufficient evidence
Organisational	Provide training and/or intervention programmes	6^17–22^	⊕⊝⊝⊝ Very low
	Provide social support	6^19,21,22,46,47,53^	Insufficient evidence
	Provide mental health support	4^19,21–23^	Insufficient evidence
	Provide personal protective equipment (PPE)	5^19–21,23,53^	⊕⊝⊝⊝ Very low
	Allow for work-life balance	3^19,20,22^	Insufficient evidence
	Allow for personal autonomy	2^19,53^	Insufficient evidence
	Effective leadership	4^19–21,47^	Insufficient evidence
Governmental	Public education about COVID-19	2^20,21^	Insufficient evidence
	Public education about personal protection	1^21^	Insufficient evidence

GRADE Working Group grades of evidence:

High certainty: We are very confident that the true effect lies close to that of the estimate of the effect.

Moderate certainty: We are moderately confident in the effect estimate; the true effect is likely to be close to the estimate of the effect, but there is a possibility that it is substantially different.

Low certainty: Our confidence in the effect estimate is limited; the true effect may be substantially different from the estimate of the effect.

Very low certainty: We have very little confidence in the effect estimate; the true effect is likely to be substantially different from the estimate of effect.

Almost none of the high-level strategies or specific programmes were evaluated for their effectiveness, within or outside of COVID-19. Moreover, only one review^24^ focused on longitudinal work, while also including and merging together non-longitudinal work, such as naturalistic studies. Indeed, 8 of the 15 reviews (53%) called for longitudinal research as future work. One exception was a significant effect of the number of protective measures and equipment provided within work contexts on reducing psychological distress, according to the reporting of Giorgi et al.^20^ on 6 of 42 papers. However, no risk of bias or quality assessment was conducted, limiting our ability to draw conclusions on the strength or generalisability of this strategy. The other exception was PFA. Pollock and colleagues^18^ reported on a cluster-randomised study by Sijbrandij et al.^25^ in which PFA was evaluated through a measure of burnout against a control (no intervention) at baseline, post-assessment, and follow-up stages with 408 participants. Results for completers and intention-to-treat groups indicated that there was no significant difference between groups or over time (95% CI). However, Pollock et al. noted risk of bias due to insufficient reporting, use of single items from a multi-item measure, and weak statistical analyses. Subsequently, we are not confident that there is sufficient evidence to draw conclusions about the efficacy of PFA. Indeed, we are not confident to recommend any of these high-level strategies or specific programmes, based on the review work so far.

## Discussion

Review work, especially systematic surveys, are considered the gold standard of evidence^13^. A wide range of professionals rely on review syntheses to make decisions on policy, practice, and research^26^. In global pandemics, psychological health and resilience are key variables that impact the ability of individuals and populations to recover and carry on. As such, recognition of resilience factors, methods of measuring resilience, and strategies to build and maintain resilience are essential. Unfortunately, this meta-review indicates that the present body of review work is severely limited, leaving us unable to confidently summarise or synthesise knowledge for public health. The implications are grave, particularly given that some of this research has already been used to inform decision-making and justify subsequent research. Additionally, it is difficult to advocate for or against measures and guidance in terms of clinical practice.

Assessing review quality is one of the main objectives of meta-review work^13^. The quality of this corpus was very low overall. Furthermore, a large portion of the work could not be assessed due to insufficiency in reporting and weaknesses in review methodology. The intended main target—strategies for psychological resilience—was particularly impacted. The narrative reviews were notably biased, characterised by opinions and claims without literature backing or reasoning. The quality of most of these reviews was subsequently too low to meet the standard for inclusion in our analyses. The other types of reviews were also insufficient to draw conclusions. Meta-analyses were not possible due to the sheer variety of measures (i.e., heterogeneity) and disconnect between these measures and the strategies reported. Indeed, we found a preponderance of instruments for a relatively short list of measures, with little reasoning behind this diversity. Moreover, most of the strategies reported were mere suggestions rather than options grounded in evidence-based sources. The two strategies that did have some evidentiary support—namely, providing PPE and training or intervention programmes—were nevertheless deemed by ourselves and the original reviewers as very low in quality. In short, we have found clear and widespread evidence that the review work on psychological resilience has been subject to the COVID-19 “paperdemic” phenomenon^15^. This leaves us unable to provide recommendations with confidence. Yet, some of these reviews, notably Preti et al.^27^ (*c* = 269), Etkind et al.^28^ (*c* = 171), and Heath et al.^29^ (*c* = 128), have received a lot of attention via citations, news outlets, and social media. In light of their quality, reliance on these papers to inform policy and practice is inadvisable. At best, these reviews signal a keen interest and urgent need for rigorous, empirical work on matters pertaining to psychological resilience.

Synthesising the nature of the review work revealed several biases and gaps. Most of the reviews were focused on frontline healthcare workers (HCWs) and women. Yet, the literature points to several other groups for whom psychological resilience and/or well-being may be integral within the context of COVID-19, including older adults^30^, people with disabilities^31^, LGBTQ + folk^32^, people with pre-existing mental health conditions^7,30^, racialized groups and ethnic minorities^33,34^, and people living in low-income and/or isolated areas^35^. A certain level of bias in focus is a natural and common feature of many areas of study^36^. Yet, it cannot be allowed to influence review work, once discovered. We encourage researchers and practitioners to consider work focused on these overlooked populations. Additionally, the way that psychological resilience has been approached needs reconsideration. We found a negative bias in factors and measures. Most measures defined resilience as the absence of mental health problems rather than the presence of fortitude, flexibility, growth, and so on. We also found a concerted focus on risk, rather than protective, factors. While identifying who may be more susceptible and in what contexts is important, it is equally important to determine what characteristics and conditions are favourable to higher rates of psychological resilience. Mental health and well-being stressors may be unavoidable in a pandemic, which this negative bias highlights. Yet, without knowledge of additive and protective factors, it is difficult to make suggestions for clinical practice. Our tables highlight these gaps and can be used to guide future research. Finally, the gap between psychological resilience measures and strategies needs to be addressed, with strategies assessed via these measures in longitudinal studies within the context of COVID-19. Clinical practice and public health would be well-served by a direct link between negative or positive outcomes and the various strategies offered. Without this work and the consensus that a review of it could offer, we cannot make recommendations with confidence.

Methodologically, there was some consistency in the limitations observed in the surveyed review work. Most reviews were not associated with a registered protocol, such as on PROSPERO or Covidence. This created undue repetition in the corpus. It is strongly encouraged that all review protocols be registered in advance; with a hot topic like COVID-19, it is likely that review work is already being undertaken. Additionally, most works included research conducted outside of the COVID-19 pandemic and did not distinguish which results were particular to COVID-19. As such, we cannot draw conclusions on whether there are any special features of the COVID-19 context relevant to psychological resilience. Future work should focus on research conducted during COVID-19 or should delineate between studies conducted during COVID-19 and other contexts, including other pandemics.

This meta-review is limited in a few ways. The heterogeneity in the corpus made it difficult to find and extract data for synthesis and comparison. For example, some reviews reported on sample size in terms of the number of people, while others reported on the number of hospitals or used another population metric. Additionally, finding a “one size fits all” tool for quality and risk of bias assessment proved challenging. This may be a matter of the topic (i.e., a feature of work on psychological resilience) or the breadth of review types included. As with most meta-reviews, included reviews sometimes reported on the same studies, and so certain characteristics that appear to be common across reviews may actually reflect multiple citations of the same study. This issue also limits the accuracy of estimating the number of studies (aggregated across the reviews) that were surveyed. While it is beyond the scope of the present work, this may be addressed by extracting the studies from all reviews, eliminating duplicates, and re-conducting the analyses for each review—a significant effort that may not yield findings equivalent in value to the time and labour required.

The original search was conducted in June 2021, and more reviews are likely to have been published since that time. A retrospective covering the “last waves” of the pandemic will be a necessary future complement to the present meta-review. In the meantime, we briefly comment on a few relevant papers that speak to the issue of longitudinal changes during the pandemic. Riehm et al.^37^ noted that time as a factor of resilience is severely understudied. Their findings from over 6000 adults in the Understanding America Study showed that mental distress varied markedly by resilience level during the early months of the COVID-19 pandemic, with low-resilience adults reporting the largest increases in mental distress. Bäuerle et al^38^. evaluated the impact of the “CoPE It” e-mental health intervention designed to improve resilience to mental distress during the pandemic. However, while they found a significant net gain between baseline and post-intervention, they relied on data obtained at only two time points and did not use a control group. There remains an urgent need for longitudinal studies of the effectiveness of interventions to increase psychological resilience during pandemics. A recently published study protocol by Godara et al.^39^ exemplifies the type of research that is needed in this area. The planned study on a mindfulness intervention would last ten weeks, involve 300 participants, include a control group, and cover a range of key outcomes, such as levels of stress, loneliness, depression and anxiety, resilience, prosocial behaviour, empathy, and compassion. This proposed study and others like it could provide the needed information on the effectiveness of interventions to improve psychological resilience that is currently lacking.

We conclude with a sober reflection on the state of affairs. As this meta-review has shown, there is insufficient high-quality evidence to inform policy and practice. The silver lining is that a way forward can be mapped through the gaps and weaknesses that characterise this body of work. We urgently recommend the following:

- Systematic reviews that follow international standards for methodology (e.g., Cochrane, JBI) and register their protocol through PROSPERO or an equivalent independent body.
- Empirical work that uses a common means of measuring positive and negative states and traits related to psychological resilience.
- Empirical work that evaluates the proposed psychological resilience strategies, including training interventions and programmes, during COVID-19.
- Empirical and review work that targets a range of population subsets beyond frontline HCWs in a broader range of geographical locations and cultural contexts.
- Empirical work that involves experimental control, longitudinal designs, naturalistic settings, and other rigorous approaches.

## Methods

We conducted a systematic meta-review of literature reviews on psychological resilience during the COVID-19 pandemic. We followed the Preferred Reporting Items for Systematic review and Meta-Analysis (PRISMA) guidelines^40^ with modifications for meta-reviews based on Aromataris et al.^13^. Our PRISMA checklist for the abstract is in Supplementary Table 1 and our PRISMA checklist for the article is in Supplementary Table 2. We used the protocol available in Seaborn et al.^6^. This protocol was registered in advance of data collection with PROSPERO on February 17, 2021 under registration ID CRD42021235288.

### Eligibility criteria

All types of reviews that summarised empirical work on psychological resilience in relation to the COVID-19 pandemic were included. We aimed to source only the highest quality of work available. As such, included reviews needed to be published in an academic or medical trade venue and peer reviewed as a basic criterion for quality. Publications from the start of the pandemic (January 2020) until the start of the review (June 2021) were included. Only reviews written in English were included, as this was the language known by all of the authors and the current international standard. Theory and opinion papers were not included, as they would not provide the type of summarised evidence sought for public health decision making. Inaccessible and unpublished literature reviews, including papers posted to archival websites and grey literature, were excluded because a minimum of quality could not be guaranteed.

### Information sources, search strategy, and study selection

Three databases, PubMed, Scopus, and Web of Science, were searched between January 16 and 19, 2021, with an update on June 9, 2021. Full search terms and queries can be found in Supplementary Table 3. A1, A3, and A2 conducted the searches, saving the results to Zotero and removing duplicates there. The combined list was then uploaded to Covidence. A1, A4, and A2 independently screened the papers in two phases: first based on the titles, keywords, and abstracts, and then based on the full text. A list of reviews excluded at the full text stage is available in Supplementary Table 4. A1 and A4 divided the work and A2 screened all papers. Conflicts were resolved by involving the other reviewer.

### Data collection and extraction

A1, A3, and A2 independently extracted data into a Google Sheet. A1 and A3 extracted data for about 50% of the total papers each, and A2 extracted data from all papers. A4 was assigned to resolve conflicts between the sets of data extractions. Data extraction variables were decided based on an extension of PICOS^41^ for meta-reviews^13^. These included: article title, authors, year of publication, objectives, type of review, participant demographics (population subset, setting), number of databases searched, date ranges of database searches, publication date ranges of reviewed articles, number of studies, types of studies, country of origin of studies, study risk of bias/quality assessment tool used, protocol registration, citation count via Google Scholar, outcomes reported, method/s of analysis, measures of psychological resilience, their instruments, whether they were tested in COVID-19, how they were assessed (i.e., statistically), CIs, measures used to evaluate strategies, their instruments, whether they were tested in COVID-19, how they were assessed (i.e., statistically), CIs, thematic frameworks, and major finding.

### Risk of bias and confidence assessments

Risk of bias and quality assessments were independently conducted by A1, A4, and A2 using a Google Form and Sheet. A1 and A4 were each responsible for about 50% of the papers, and A2 assessed all papers. We used the Scale for the Assessment of Narrative Review Articles (SANRA)^42^ for qualitative reviews and the JBI Critical Appraisal Checklist for Systematic Reviews and Research Synthesis^13^ for the rest. In contrast to the protocol, we did not use the AMSTAR-2 because there were too few reviews that met the characteristics required for that tool. Sums were averaged across the reviewers. Cut-offs were determined after evaluating and comparing the reviews in a weighted fashion; for both, the cut-off was set at 0.8. Only the data of reviews that met the standard of quality were used to answer RQ2. Confidence in the quality of evidence was assessed by A1 and A2 using the Grading of Recommendations Assessment, Development, and Evaluation (GRADE) system^43^.

### Data analysis

The planned meta-synthesis could not be conducted due to the nature of the reviews captured. As such, measures of effect, variability (i.e., heterogeneity), and other inferential statistics could not be generated. Instead, a combination of descriptive statistics and thematic analyses were generated to identify meaningful patterns across the data^44,45^. A3 was responsible for the descriptives. A1, A4, and A2 conducted the thematic analyses. High-level themes were inductively derived as a means of “seeing across” the corpus of review work, while most sub-themes were semantically derived, using the words found within the reviews. All thematic analyses involved a standard, rigorous process of familiarisation with the data, initial coding by one reviewer, generation of initial themes by that reviewer, independent application of those themes by two reviewers, discussion and re-review until conflicts were resolved or themes discarded, and finalisation of themes by the first reviewer. A4 was the first reviewer for the measures data. A1 was the first reviewer for the strategies and risk/protective factors data. A2 was the second reviewer in all cases. We used Google Sheets for all analyses.

## Supplementary information

Supplementary Information

## Data Availability

Most of the data is included in this paper and/or the Supplementary Information. All other data can be made available by the authors upon request.
