# Supplementary material for: A meta-review of psychological resilience during COVID-19
Source: Npj Ment Health Res. 2022 Jul 5;1:5. doi: 10.1038/s44184-022-00005-8 (PMC9255496; doi:10.1038/s44184-022-00005-8)
Supplement: Supplementary file 1 — Supplementary Information [file 44184_2022_5_MOESM1_ESM.pdf]

Supplementary Information

Supplementary Table 1. PRISMA 2020 for Abstracts Checklist

From: Page MJ, McKenzie JE, Bossuyt PM, Boutron I, Hoffmann TC, Mulrow CD, et al. The PRISMA 2020 statement: an updated guideline for reporting systematic reviews. BMJ 2021;372:n71. doi: 10.1136/bmj.n71

Section and Topic	Item #	Checklist item	Reported (Yes/No)
**TITLE**			
Title	1	Identify the report as a systematic review.	Yes
**BACKGROUND**			
Objectives	2	Provide an explicit statement of the main objective(s) or question(s) the review addresses.	Yes
**METHODS**			
Eligibility criteria	3	Specify the inclusion and exclusion criteria for the review.	Yes
Information sources	4	Specify the information sources (e.g. databases, registers) used to identify studies and the date when each was last searched.	Yes
Risk of bias	5	Specify the methods used to assess risk of bias in the included studies.	Yes
Synthesis of results	6	Specify the methods used to present and synthesise results.	Yes
**RESULTS**			
Included studies	7	Give the total number of included studies and participants and summarise relevant characteristics of studies.	Yes
Synthesis of results	8	Present results for main outcomes, preferably indicating the number of included studies and participants for each. If meta-analysis was done, report the summary estimate and confidence/credible interval. If comparing groups, indicate the direction of the effect (i.e. which group is favoured).	Yes
**DISCUSSION**			
Limitations of evidence	9	Provide a brief summary of the limitations of the evidence included in the review (e.g. study risk of bias, inconsistency and imprecision).	Yes
Interpretation	10	Provide a general interpretation of the results and important implications.	Yes
**OTHER**			
Funding	11	Specify the primary source of funding for the review.	Yes
Registration	12	Provide the register name and registration number.	Yes

Supplementary Table 2. PRISMA Checklist

From: Page MJ, McKenzie JE, Bossuyt PM, Boutron I, Hoffmann TC, Mulrow CD, et al. The PRISMA 2020 statement: an updated guideline for reporting systematic reviews. BMJ 2021;372:n71. doi: 10.1136/bmj.n71

Section and Topic	Item #	Checklist item	Location where item is reported
**TITLE**			
Title	1	Identify the report as a systematic review.	Page 1
**ABSTRACT**			
Abstract	2	See the PRISMA 2020 for Abstracts checklist.	Page 2
**INTRODUCTION**			
Rationale	3	Describe the rationale for the review in the context of existing knowledge.	Page 3
Objectives	4	Provide an explicit statement of the objective(s) or question(s) the review addresses.	Page 3
**METHODS**			
Eligibility criteria	5	Specify the inclusion and exclusion criteria for the review and how studies were grouped for the syntheses.	Pages 14-15
Information sources	6	Specify all databases, registers, websites, organisations, reference lists and other sources searched or consulted to identify studies. Specify the date when each source was last searched or consulted.	Page 15, Supplementary Table 3
Search strategy	7	Present the full search strategies for all databases, registers and websites, including any filters and limits used.	Page 15, Supplementary Table 3
Selection process	8	Specify the methods used to decide whether a study met the inclusion criteria of the review, including how many reviewers screened each record and each report retrieved, whether they worked independently, and if applicable, details of automation tools used in the process.	Page 15
Data collection process	9	Specify the methods used to collect data from reports, including how many reviewers collected data from each report, whether they worked independently, any processes for obtaining or confirming data from study investigators, and if applicable, details of automation tools used in the process.	Page 15
Data items	10a	List and define all outcomes for which data were sought. Specify whether all results that were compatible with each outcome domain in each study were sought (e.g. for all measures, time points, analyses), and if not, the methods used to decide which results to collect.	Page 15
	10b	List and define all other variables for which data were sought (e.g. participant and intervention characteristics, funding sources). Describe any assumptions made about any missing or unclear information.	Page 15
Study risk of bias assessment	11	Specify the methods used to assess risk of bias in the included studies, including details of the tool(s) used, how many reviewers assessed each study and whether they worked independently, and if applicable, details of automation tools used in the process.	Pages 15-16
Effect measures	12	Specify for each outcome the effect measure(s) (e.g. risk ratio, mean difference) used in the synthesis or presentation of results.	Page 16
Synthesis methods	13a	Describe the processes used to decide which studies were eligible for each synthesis (e.g. tabulating the study intervention characteristics and comparing against the planned groups for each synthesis (item #5)).	n/a
	13b	Describe any methods required to prepare the data for presentation or synthesis, such as handling of missing summary statistics, or data conversions.	Page 16
	13c	Describe any methods used to tabulate or visually display results of individual studies and syntheses.	n/a
	13d	Describe any methods used to synthesize results and provide a rationale for the choice(s). If meta-analysis was performed, describe the model(s), method(s) to identify the presence and extent of statistical heterogeneity, and software package(s) used.	Page 16
	13e	Describe any methods used to explore possible causes of heterogeneity among study results (e.g. subgroup analysis, meta-regression).	n/a
	13f	Describe any sensitivity analyses conducted to assess robustness of the synthesized results.	n/a

Section and Topic	Item #	Checklist item	Location where item is reported
Reporting bias assessment	14	Describe any methods used to assess risk of bias due to missing results in a synthesis (arising from reporting biases).	Pages 15-16
Certainty assessment	15	Describe any methods used to assess certainty (or confidence) in the body of evidence for an outcome.	Page 16
**RESULTS**			
Study selection	16a	Describe the results of the search and selection process, from the number of records identified in the search to the number of studies included in the review, ideally using a flow diagram.	Page 4
	16b	Cite studies that might appear to meet the inclusion criteria, but which were excluded, and explain why they were excluded.	Page 4, Supplementary Table 4
Study characteristics	17	Cite each included study and present its characteristics.	Page 5, Supplementary Table 5
Risk of bias in studies	18	Present assessments of risk of bias for each included study.	Pages 5-6
Results of individual studies	19	For all outcomes, present, for each study: (a) summary statistics for each group (where appropriate) and (b) an effect estimate and its precision (e.g. confidence/credible interval), ideally using structured tables or plots.	Pages 7-12
Results of syntheses	20a	For each synthesis, briefly summarise the characteristics and risk of bias among contributing studies.	n/a
	20b	Present results of all statistical syntheses conducted. If meta-analysis was done, present for each the summary estimate and its precision (e.g. confidence/credible interval) and measures of statistical heterogeneity. If comparing groups, describe the direction of the effect.	Pages 7-12
	20c	Present results of all investigations of possible causes of heterogeneity among study results.	n/a
	20d	Present results of all sensitivity analyses conducted to assess the robustness of the synthesized results.	n/a
Reporting biases	21	Present assessments of risk of bias due to missing results (arising from reporting biases) for each synthesis assessed.	n/a
Certainty of evidence	22	Present assessments of certainty (or confidence) in the body of evidence for each outcome assessed.	Page 7, Pages 9-11
**DISCUSSION**			
Discussion	23a	Provide a general interpretation of the results in the context of other evidence.	Page 12
	23b	Discuss any limitations of the evidence included in the review.	Pages 12-13
	23c	Discuss any limitations of the review processes used.	Pages 13-14
	23d	Discuss implications of the results for practice, policy, and future research.	Page 14
**OTHER INFORMATION**			
Registration and protocol	24a	Provide registration information for the review, including register name and registration number, or state that the review was not registered.	Page 14
	24b	Indicate where the review protocol can be accessed, or state that a protocol was not prepared.	Page 14
	24c	Describe and explain any amendments to information provided at registration or in the protocol.	Pages 15-16
Support	25	Describe sources of financial or non-financial support for the review, and the role of the funders or sponsors in the review.	Page 19
Competing interests	26	Declare any competing interests of review authors.	Page 20
Availability of data, code and other materials	27	Report which of the following are publicly available and where they can be found: template data collection forms; data extracted from included studies; data used for all analyses; analytic code; any other materials used in the review.	Page 20

Supplementary Table 3. Search queries with full search terms for all databases

Database	Metadata	Query	Filters
Web of Science	Topic (TS)	(AB=resilien* AND TS=(resilien* OR psycholog* OR personal* OR positiv* OR "cope" OR "coping" OR "personal strength") AND TS=((covid OR covid-19 OR coronavirus OR Sars-Cov-2) AND ("literature review" OR "systematic survey" OR "systematic review" OR "rapid review" OR "literature survey" OR "review article" OR "survey article" OR "meta-analysis" OR "living review" or "mapping review" or "qualitative evidence synthesis" or "scoping review" or "state-of-the-art review" or "systematized review" or "narrative review" or "comprehensive review")))	Year (2020, 2021), Language (English), Doctype (Article, Book, Book Chapter, Early Access, Proceedings Paper)
Scopus	Article title, Abstract, Keywords	(TITLE-ABS(resilien*) AND (TITLE-ABS-KEY(psycholog*) OR TITLE-ABS-KEY(personal*) OR TITLE-ABS-KEY(positiv*) OR TITLE-ABS-KEY(cope) OR TITLE-ABS-KEY(coping) OR TITLE-ABS-KEY("personal strength")) AND (TITLE-ABS-KEY(covid) OR TITLE-ABS-KEY(covid-19) OR TITLE-ABS-KEY(coronavirus) OR TITLE-ABS-KEY(sars-cov-2)) AND (TITLE-ABS-KEY("literature review") OR TITLE-ABS-KEY("systematic survey") OR TITLE-ABS-KEY("systematic review") OR TITLE-ABS-KEY("rapid review") OR TITLE-ABS-KEY("literature survey") OR TITLE-ABS-KEY("review article") OR TITLE-ABS-KEY("survey article") OR TITLE-ABS-KEY("meta-analysis") OR TITLE-ABS-KEY("living review") OR TITLE-ABS-KEY("mapping review") OR TITLE-ABS-KEY("qualitative evidence synthesis") OR TITLE-ABS-KEY("scoping review") OR TITLE-ABS-KEY("state-of-the-art review") OR TITLE-ABS-KEY("systematized review") OR TITLE-ABS-KEY("narrative review") OR TITLE-ABS-KEY("comprehensive review")))	Year (2020, 2021), Language (English)
PubMed	Title/Abstract, Text Word	((resilien*[Title/Abstract]) AND (resilien*[Text Word] OR psycholog*[Text Word] OR personal*[Text Word] OR positiv*[Text Word] OR cope[Text Word] OR coping[Text Word] OR "personal strength"[Text Word]) AND (SARS-CoV-2[Text Word] OR COVID-19[Text Word] OR Coronavirus[Text Word]) AND ("literature review"[Text Word] OR "systematic survey"[Text Word] OR "systematic review"[Text Word] OR "rapid review"[Text Word] OR "literature survey"[Text Word] OR "review article"[Text Word] OR "survey article"[Text Word] OR "meta-analysis"[Text Word] OR "living review"[Text Word] OR "mapping review"[Text Word] OR "qualitative evidence synthesis"[Text Word] OR "scoping review" [Text Word] OR "state-of-the-art review"[Text Word] OR "systematized review"[Text Word] OR "narrative review"[Text Word] OR "comprehensive review"[Text Word]))	Year (2020, 2021), Language (English)

Supplementary Table 4. List of reviews excluded at the full-text screening stage

Article Title	Exclusion Reason
Volkan and Volkan (2020)	Not a review
Sriharan et al. (2021)	Not psychological resilience
Raudenská et al. (2020)	Not psychological resilience
Rajkumar (2020)	Not psychological resilience
Mitchinson et al. (2021)	Not a review
Michie et al. (2020)	Not psychological resilience
Della Monica et al. (2021)	Not psychological resilience
Fegert et al. (2020)	Not psychological resilience
Mitchell et al. (2020)	Not psychological resilience
Marler and Ditton (2021)	Not psychological resilience
Karimi et al. (2020)	Not English
Diaz et al. (2021)	Not psychological resilience
Caponnetto et al. (2021)	Not psychological resilience
Abdoul-Azize and El Gamil (2020)	Not psychological resilience
Castellini et al. (2020)	Wrong study design
El-Hage et al. (2020)	Not English
Chastin et al. (2021)	Not psychological resilience
Bohlken et al. (2020)	Not English
Daly and Robinson (2021)	Wrong study design
Fouché et al. (2020)	Wrong study design
Black et al. (2020)	Not psychological resilience

Supplementary Table 5. Full summary table of included reviews

	Type of Review	Population	Setting and/or Context	Country of Origin of Studies	Number of Databases	Date Range of Search	Publication Date Range of Studies	Number of Studies	Types of Studies (Number of Studies of Type)
Jans-Beken (2021)	Narrative	..	..	..	..	..	..	..	..
De Kock et al. (2021)	Rapid	Frontline medical and dental workers, hospital and medical admin staff	Hospitals, clinics, medical centres	China, America, Israel, UK, Singapore, Pakistan, Singapore, India, global	14	April 23, 2020 - May 6, 2020 (one on April date and again on May date)	1946-2020	24	- cross-sectional (18)<br>- mixed methods (2)<br>- qualitative (2)<br>- longitudinal (1)<br>- uncontrolled before-after study (1)
Schwartz et al. (2020)	Narrative	Healthcare workers (HCW): clinicians, nurses, physicians, frontline workers	..	..	4	May 26, 2020	July 2004 - May 2020	96	..
Blanca et al. (2020)	Rapid	HCW, patients	..	..	..	..	..	..	..
Hooper et al. (2021)	Systematic	Frontline responders, emergency medical first responders, forensic personnel, first responders in active duty, Ebola medical providers, active full-time firefighters, HCW, social service workers, army personnel, military personnel, police officers, marines	Ebola areas in West Africa, Australia, human massacres, mass gatherings, military deployment, car crash fatalities, maritime collisions, Afghanistan, natural disasters, mass shooting events, other various local disasters, social service and disaster response organisations	Hong Kong, USA, Mexico, West Africa, Australia, Afghanistan (on British participants), UK	4	July 2020	2005 - 2020	12	- RCT (3)<br>- cluster RCT (2)<br>- cross-sectional (4)<br>- quasi- experimental designs (3)

Giorgi et al. (2020)	Narrative	HCW, frontline workers/staff, migrant workers, vulnerable groups, professionals, industrial service, personnel, employees	Workplace, organisation, job, job task, occupation, occupational	Iran, Japan, India, China, USA, Italy, Latin America	3	July 2020	December 2019 - July 2020	37	- narrative review (4)<br>- cross sectional survey (26)<br>- perspective/lit review (2)<br>- position paper (1)<br>- brief report (1)<br>- commentary (1)<br>- descriptive study (1)<br>- case study (1)
Wright et al. (2021)	Scoping	Perinatal clinicians including midwives and certified nurse-midwives, hospital maternity staff (support workers, student midwives, doctors, lecturers, community, hospital, and research midwives)	Hospital, anywhere with internet access	UK, USA	4	-	January 2015 - January 2020	2	- qualitative study using questionnaires (2)
Varghese et al. (2021)	Systematic	Nurses, other healthcare professionals	Hospitals	Brazil, Croatia, Germany, Poland, Russia, Italy, Jordan, China, Vietnam, Turkey, Singapore, Philippines, Oman, Iran, India	8	October 5, 2020	March 11, 2020 - October 5, 2020	26	- cross sectional study (19)<br>- cross sectional survey (7)
Sterina et al. (2021)	Systematic	Older adults	..	China, Spain, USA, Italy, Taiwan, Algeria, Canada, UK	7	June 2020	2000 - June 2020	20	..
De Brier et al. (2020)	Rapid	HCW, administrative personnel, nurses, volunteers, medical staff, infected HCW, high-risk HCW, hospital employees, frontline HCWs, doctors	Hospital, health centre, community health network	Taiwan, Singapore, Hong Kong, China, South Korea, Canada	3 + COVID-19 resource	Databases: March 24, 2020; COVID-19 resource: May 28, 2020	2004-2020	33	- cross-sectional observational studies (32)<br>- uncontrolled before-and-after (1)
Sirois and Owens (2021)	Rapid	HCW, nurses, physicians, doctors, midwives, HCW-ancillary workers, allied health professionals, technicians (radiology staff),	Hospital settings	Kurdistan, China, Turkey, Oman, Egypt, Saudi Arabia, Pakistan, Italy, England, Greece, France, Singapore, India, Taiwan, Brazil,	4	April 6, 2020, then with updates on June 7, July 2, July 10, July 30,	2000 - November 15, 2020	139	- cross-sectional observational studies (136)<br>- cross sectional study at 2 time points (1)

		anaesthesiologists, medical staff and students, medics, ED nurses, frontline hospital HCW		Spain, USA, Australia, Libya, Malaysia, Canada, Argentina, Hong Kong, Iran, Sierra Leone, South Korea, Palestine, Japan, Israel, Mali, Jordan, Russia, Serbia, South Ethiopia		November 15, 2020			- prospective study (1)
Rieckert et al. (2021)	Scoping	HCW, physicians, nurses	Hospital settings	Saudi Arabia, Sierra Leone, Taiwan, Netherlands, Liberia, Canada, Spain, Singapore, China, Congo, Uganda, Japan, South Korea, Germany, USA	4 + 2 grey	March 26, 2020	January 1, 2003 - March 26, 2020; grey literature on bioRxiv and medRxiv: January 3, 2020 - March 26, 2020	71 + 5 grey	- quantitative (43)<br>- qualitative (21)<br>- mixed-methods (4)<br>- systematic review (2)<br>- focused review (1)
Hughes et al. (2021)	Rapid	Informal or family caregivers, informal caregivers of people with dementia, individuals with a (major) neurocognitive disorder, individuals with Alzheimer's disease	..	Italy, Greece, Portugal, India, Argentina, England	5	March 15, 2021	2020 - 2021	10	- 10 quantitative studies including surveys administered over the phone or online
Pollock et al. (2020)	Systematic	HCW: nurses, midwives, mental health clinicians, social workers, nursing students, physicians, medical assistants, ICU staff, medical workers, doctors; volunteers, burial teams, administrators, technicians, teachers, caregivers; non-professionals - cleaning and security staff	University, hospital, medical centre, clinic, community, ICU, ambulance services, treatment centre	Netherlands, UK, USA, China, Spain, Taiwan, Liberia, Sierra Leone, West Africa, South Korea	8	May 28, 2020	2002 - May 28, 2020	16	- qualitative (7)<br>- descriptive (9)
Batra et al. (2020)	Systematic	Nurses, physicians or doctors, allied health staff, laboratory	..	China, India, Singapore, Iran, Pakistan, Jordan,	5 + preprints	July 27, 2020	December 1, 2019 - July 27, 2020	65	- cross sectional observational studies (65)

		specialists, anesthetist technicians and general technicians, physical therapists, pharmacists, dental professionals		Bahrain, Hong Kong, Israel, Nepal, Oman, Saudi Arabia, South Korea, Italy, Turkey, Switzerland, Serbia, Ireland, Argentina, Brazil, Chile, Mexico, USA					
Kunzler et al. (2021)	Scoping	General and subsets: hospital staff, HCW, quarantined patients/family and medical staff, hospital workers (all employees and professional staff), emergency hotline workers, individuals deployed by the CDC, Individuals before deployment by the CDC, Ebola treatment centre staff, non-specialist volunteers, adolescents at junior high school, children (Ebola survivors, from Ebola-infected homes, living in Ebola-affected community), town community members, home-quarantined college students, Ebola patients, people with chronic diseases	..	Canada, West Africa, China, Sierra Leone, USA, Liberia, Hong Kong, Taiwan, South Korea	3 + other	May 7, 2020	January 2000 - May 7, 2020	19	- trials including parallel controls (3): randomised study (1), RCT (1), cluster-RCT (1)<br>- single-group (11): interview study (1)<br>- case reports/studies (4)<br>- two-group study (delayed treatment model) (1)
Davis et al. (2021)	Narrative	People with multiple sclerosis, healthcare providers (HCP)	..	..	..	..	..	..	..
Chew et al. (2020)	Rapid	Nurses, physicians, survivors, doctors, health care assistants (HCA), HCW, medical staff), hospital employees, midwives, members of local councils, health facilities, and international partners,	..	West Africa, Sierra Leone, Saudi Arabia, China, Taiwan, Canada, Hong Kong, Singapore	3	April 20, 2020	Start of database - April 20, 2020	23	- cross sectional, observational (22)<br>- longitudinal, observational (1)

		multidisciplinary team (defense/ military personnel), frontline staff							
Labrague (2021)	Systematic	HCW: nurses, doctors, nursing assistants, midwives, radiologists, physiotherapists, pharmacists, health care assistants, psychologists, medical staff, physicians, technicians, health administrators, general practitioners	··	China, Italy, Philippines, USA, Turkey, Spain, Israel, Palestine, Pakistan, Egypt	5	August 2020 - October 2020	"during COVID"	31	- cross-sectional (30)<br>- longitudinal (1)
Balcombe and De Leo (2020)	Mini	(Elite) athletes	··	··	2	··	"generally from within the last 4 years"	··	- pilot, longitudinal cohort, prevalence, qualitative, monitoring
Gilan et al. (2020)	Systematic	General population, physicians, HCW, nursing staff, students, administrative staff	··	China, India, Germany, multinational China, Hong Kong, Taiwan, Macao	3	April 16, 2020	January 1, 2019 - April 16, 2020	18	- cross-sectional, survey (16)<br>- longitudinal, survey (1)
Heath et al. (2020)	Narrative	HCW	Workplace	··	··	··	··	··	··
Berger et al. (2021)	Systematic	Children and adolescents	Hospitals during outbreaks	Hong Kong, Italy, Canada, Spain/Italy, UK, multinational: US, Mexico, Canada	4	··	2003-2020	11	- retrospective cohort (1)<br>- qualitative (1)<br>- prospective cohort (3)<br>- prospective observational (1)<br>- COVID-19 studies: prospective (2)<br>- case-control (1)<br>- mixed method (1)
Ho et al. (2020)	Scoping	Medical students	··	··	6	November 17, 2019 and April 24, 2020	January 1, 2000 - March 31, 2020	52	- quantitative, qualitative
Muller et al. (2020)	Rapid	Health care workers, clinical administration, doctors, emergency	··	China, France, Germany, India, Iran, Italy, Singapore, USA	3	March 12, 2020 - May 11, 2020	Pubmed: December 1, 2019 - May 3, 2020	59	- survey (46)<br>- interviews (5)<br>- cohort/longitudinal (2)

		staff, medical students, nurses					Embase: December 1, 2019 - March 27, 2020<br>CDC: December 1, 2019 - May 11, 2020		- systematic review (2)<br>- other (4) - review of online surveys, case studies, described up-take of an intervention
Kaur and Som (2020)	Narrative	··	··	··	··	··	··	··	··
Prat and Mancini (2021)	Rapid	General population	Pre- and post-COVID-19 lockdowns	Continents: Asia, North America, Oceania, Europe	5	June 2020	January 2020 - June 2020	25	- longitudinal studies (12)<br>- natural experiments (13)
Preti et al. (2020)	Rapid	HCW: physicians, nurses, and auxiliaries; staff members: admin workers and technicians	··	China, Canada, Taiwan, South Korea, Saudi Arabia, Greece, Nigeria, Sierra Leone, Liberia, Singapore, Japan	3	March 30, 2020	2004-2020	44	- cross-sectional (34)<br>- longitudinal (2)<br>- cross-sectional and case-control (6)<br>- longitudinal and case-control (2)
Seifert et al. (2020)	Qualitative	··	··	··	1	July 24, 2020 - October 29, 2020	October 29, 2020	··	··
Etkind et al. (2020)	Rapid	Palliative care and hospice workers, clinicians, decision makers, physicians, nurses, disaster management and public health experts, social workers, pharmacists	Hospice, hospital, palliative care unit, community care centres	Italy, West Africa, Sierra Leone, Hong Kong, US, Taiwan, Singapore	5	March 18, 2020	2004 - March 18, 2020	10	- qualitative interviews (3)<br>- cross-sectional survey (2)<br>- case report (1)<br>- case study (1)<br>- cross sectional assessment (1)<br>- simulation exercises with recommendations (1)<br>- retrospective study using administrative data (1)
